# Diversity of *Plasmodium falciparum* Chloroquine Resistance Transporter (*pfcrt*) Exon 2 Haplotypes in the Pacific from 1959 to 1979

**DOI:** 10.1371/journal.pone.0030213

**Published:** 2012-01-17

**Authors:** Chim W. Chan, Rita Spathis, Dana M. Reiff, Stacy E. McGrath, Ralph M. Garruto, J. Koji Lum

**Affiliations:** 1 Laboratory of Evolutionary Anthropology and Health, Binghamton University, Binghamton, New York, United States of America; 2 Laboratory of Biomedical Anthropology and Neurosciences, Binghamton University, Binghamton, New York, United States of America; 3 Department of Anthropology, Binghamton University, Binghamton, New York, United States of America; 4 Department of Biological Sciences, Binghamton University, Binghamton, New York, United States of America; Weill Cornell Medical College, United States of America

## Abstract

Nearly one million deaths are attributed to malaria every year. Recent reports of multi-drug treatment failure of *falciparum* malaria underscore the need to understand the molecular basis of drug resistance. Multiple mutations in the *Plasmodium falciparum* chloroquine resistance transporter (*pfcrt*) are involved in chloroquine resistance, but the evolution of complex haplotypes is not yet well understood. Using over 4,500 archival human serum specimens collected from 19 Pacific populations between 1959 and 1979, the period including and just prior to the appearance of chloroquine treatment failure in the Pacific, we PCR-amplified and sequenced a portion of the *pfcrt* exon 2 from 771 *P. falciparum*-infected individuals to explore the spatial and temporal variation in *falciparum* malaria prevalence and the evolution of chloroquine resistance. In the Pacific, the prevalence of *P. falciparum* varied considerably across ecological zones. On the island of New Guinea, the decreases in prevalence of *P. falciparum* in coastal, high-transmission areas over time were contrasted by the increase in prevalence during the same period in the highlands, where transmission was intermittent. We found 78 unique *pfcrt* haplotypes consisting of 34 amino acid substitutions and 28 synonymous mutations. More importantly, two *pfcrt* mutations (N75D and K76T) implicated in chloroquine resistance were present in parasites from New Hebrides (now Vanuatu) eight years before the first report of treatment failure. Our results also revealed unexpectedly high levels of genetic diversity in *pfcrt* exon 2 prior to the historical chloroquine resistance selective sweep, particularly in areas where disease burden was relatively low. In the Pacific, parasite genetic isolation, as well as host acquired immune status and genetic resistance to malaria, were important contributors to the evolution of chloroquine resistance in *P. falciparum*.

## Introduction

Malaria caused approximately 243 million clinical cases and 863,000 deaths in 2008 [1]. The development of resistance to chloroquine (CQ), once the mainstay of malaria treatment worldwide, has exacerbated malaria morbidity and mortality over the last few decades, especially in sub-Saharan Africa [2], [3]. An understanding of the evolution of CQ resistance in the malaria parasite *Plasmodium falciparum* is crucial to providing important insights into the mechanisms by which parasites respond to other aminoquinolines such as amodiaquine, which are currently used as partner drugs with artesunate in artemisinin-based combination therapies (ACTs). Such insights are urgently needed, given recent evidence of diminished efficacies of ACTs in Southeast Asia [4].

CQ resistant (CQR) *P. falciparum* show diminished accumulation of CQ in the digestive vacuole (DV), and this phenotype is correlated with amino acid substitutions in the DV membrane transporter, *P. falciparum* chloroquine resistance transporter (*pf*CRT) [5]. In *pf*CRT, the lysine to threonine substitution at codon position 76 (K76T) shows the most consistent correlation with CQ resistance, but as many as eight other amino acid substitutions are also involved in distinguishing CQR from CQ sensitive (CQS) parasites [5], [6]. Together with K76T, substitutions at positions 72, 74, and 75 form complex CQR *pfcrt* exon 2 haplotypes that correspond to the geographical origins of CQ resistance [6]. However, it is unclear how these CQR *pfcrt* haplotypes arose due to the lack of previous genetic characterization of parasites during the periods of CQ resistance emergence.

Multiple origins of CQ resistance in *P. falciparum* have been suggested based on epidemiological data [7] and genetic analyses [5], [6]. CQ treatment failures first appeared simultaneously near the Thai-Cambodian border and in Colombia in the late 1950s, and CQR parasites spread from these original foci to neighboring areas [7]. Characterization of *pfcrt* haplotypes from laboratory-adapted *P. falciparum* clones provided strong support for these two independent origins of CQ resistance, and the invasion of CQR parasites from Southeast Asia to Africa [5], [6].

In Papua New Guinea (PNG), CQ treatment failure was first reported in 1976, and was initially assumed to indicate the expansion of CQR *P. falciparum* from Southeast Asia into the Pacific [7], [8]. Examination of *pfcrt* polymorphisms showed that despite the geographic proximity to the Southeast Asian focus of resistance, CQR parasites from PNG harbored a *pfcrt* exon 2 haplotype similar to one from South America [9]. Since these *pfcrt* substitutions were associated with a different genetic background [6], [10], it was argued that PNG represented another independent focus of CQ resistance [9].

In other malarious regions of the Pacific, the evolution of CQ resistance in *P. falciparum* might have been more complex. Nagesha et al. [11] identified four *pfcrt* haplotypes associated with CQ resistance in field isolates from Indonesian Papua (West New Guinea or WNG). These haplotypes were representative of both the Southeast Asian/African (codon 72–76: CVIET) and the PNG/South American (codon 72–76: SVMNT) CQR haplotypes, and a third composite (codon 72–76: SVIET) haplotype. This region of the Pacific is also characterized by very diverse ecologies which affect parasite population dynamics that in turn play a crucial role in the evolution of CQR parasites and their dispersals [12]. In this regard, it is important to include parasites from multiple locales and examine their interactions at a population level over time.

Archival biological specimens have been shown to be useful in studying past evolutionary events such as CQ resistance in *P. falciparum* [13], [14]. We reasoned that blood samples collected prior to (and slightly after) the mid-1970s from populations residing in the malarious Pacific might contain DNA from *P. falciparum* that was under increasing CQ selection. Genotyping of *pfcrt* might therefore provide important insights into the evolution of the complex CQR haplotypes. Additionally, by including samples from multiple locations collected at various time periods, we sought to examine the spatial and temporal dynamics of the evolution and spread of CQR *P. falciparum* across the diverse environments in the Pacific [12]. Results from our study indicated that *pfcrt* exon 2 was highly diverse prior to the CQ selective sweep, and suggested that CQ resistance in the Pacific evolved *in situ* and arose first in low transmission areas, where host populations had minimal genetic resistance and acquired immunity against malaria infections.

## Results

**Figure 1:**
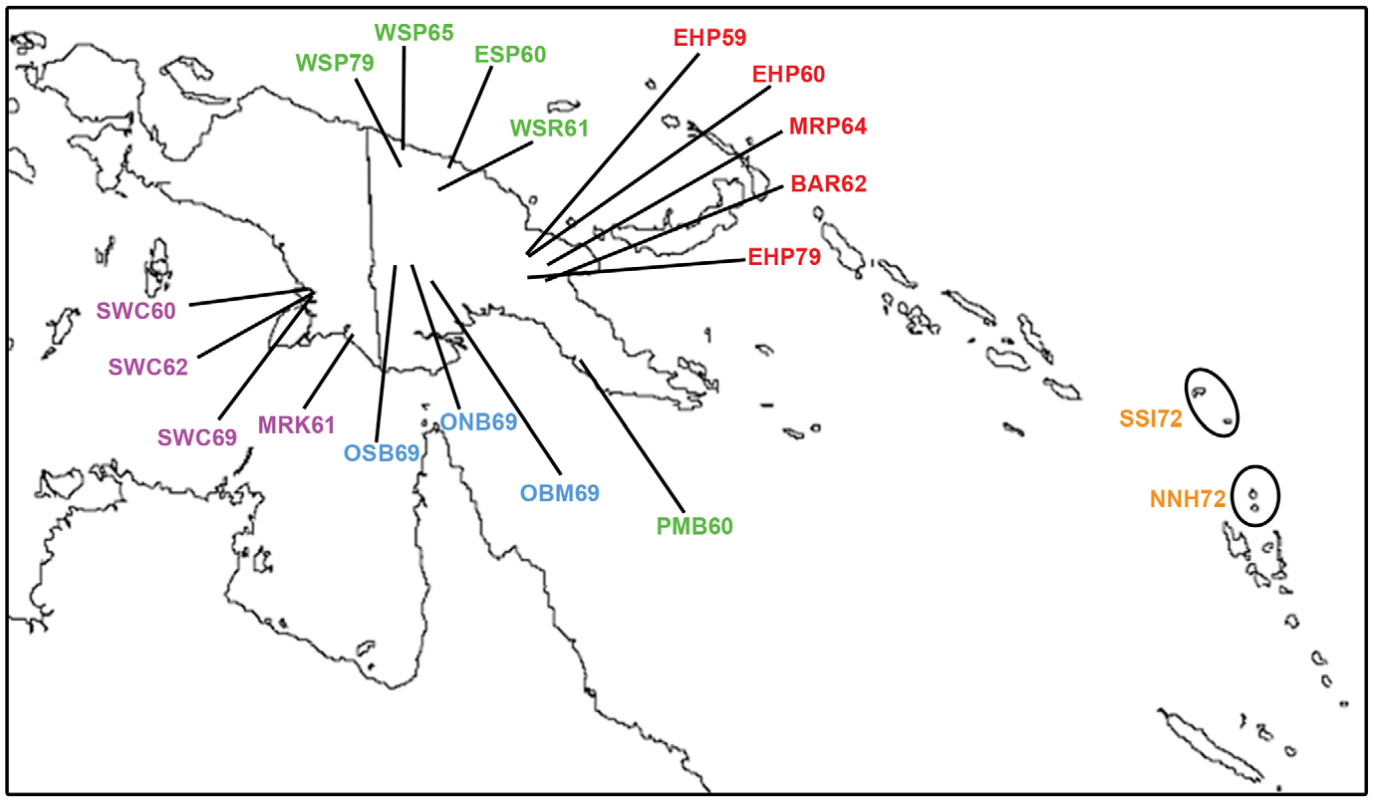
Approximate locations of populations. Populations are denoted by their sample codes (Table S1) and assigned to one of five ecological zones as indicated by colors as followed: WNG coast (purple), PNG coasts (green), PNG Papuan Plateau (blue), PNG eastern highlands (red), and Island Melanesia (orange). Samples collected from the same locations in different years (e.g. SWC60, SWC62, and SWC69) did not represent repeated samplings from the same populations.

Overall, 771 of 4598 (16.8%) specimens from 19 populations were positive for *P. falciparum* (Figure 1, Table S1), as determined by successful PCR amplification and DNA sequencing of a 195-bp segment of *pfcrt* exon 2 corresponding to codon positions 36 to 99. Rates of *P. falciparum* infection ranged from 0% in the Papua New Guinea (PNG) eastern highlands sample of BAR62 and the West New Guinea (WNG) coastal sample of MRK61 to 55.4% in the WNG coastal sample of SWC60 (Figure 1, Table S1). When analyzed by ecological zones, coastal populations from both WNG and PNG showed the highest average rates of *P. falciparum* infection at 26.9% and 20.3%, respectively, followed by those from Island Melanesia at 17.4% and PNG Papuan Plateau at 13.6%, with those from the PNG eastern highlands showing the lowest rate at 3.14% (Table S1). Our results from PNG were consistent with the observation that malaria prevalence decreases with increasing altitude [15].

**Figure 2:**
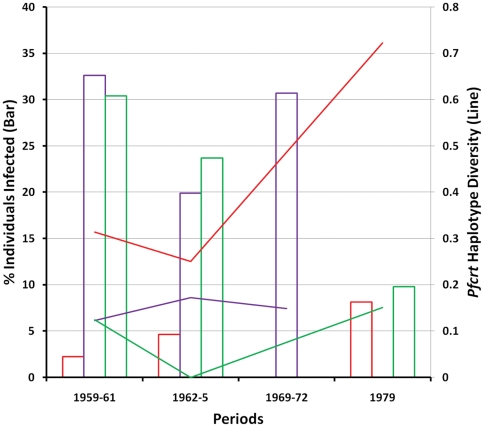
Temporal variation in *P. falciparum* infection rates (bar) and *pfcrt* haplotype diversities (line). Ecological zones are indicated by colors as followed: PNG eastern highlands (red), WNG coast (purple), and PNG coasts (green).

For three of the five ecological zones, longitudinal samples allowed for crude estimates of temporal variation in *P. falciparum* prevalences. On the WNG coast, the infection rates remained high from 1960 to 1969, while on the PNG coasts, there was a steady decrease in prevalence from 1960 to 1979. In contrast, *P. falciparum* prevalence increased slightly over the same period in the PNG eastern highlands (Figure 2).

Approximately 8.95% (69/771) of *P. falciparum*-positive specimens showed two distinct *pfcrt* exon 2 genotypes, yielding a total of 840 sequences (GenBank accession numbers HM019533–HM020128, HM020130–HM020332, and HM202334–HM020374). Samples from the PNG eastern highlands and Island Melanesia showed the highest frequencies of individuals with multiple infections (Table S1). Seventy eight unique *pfcrt* haplotypes were identified, of which the wildtype *pfcrt* sequence accounted for 83.8% (704/840) of sequences.

We calculated *pfcrt* haplotye diversities to compare the amounts of genetic variation among populations and ecological zones. Haplotype diversity indices range between 0 and 1. A measure of 0 indicates that all haplotypes within a population or ecological zone are identical to one another, whereas a measure of 1 indicates that all haplotypes within a population or ecological zone are unique. When analyzed by individual populations, *pfcrt* haplotype diversity ranged from 0 in EHP60, WSP65, and ONB69, to 0.722 in EHP79 (Table S1). When grouped into ecological zones, populations from the PNG eastern highlands and Island Melanesia had higher haplotype diversities than those from the other ecological zones, although the difference was not statistically significant (ANOVA; *p* = 0.106) (Table S1). *Pfcrt* haplotype diversities did not vary substantially over time along the WNG coast, but in both PNG coasts and eastern highlands the levels of diversity showed substantial fluctuations, with an appreciable increase over time in the PNG eastern highlands (Figure 2).

**Figure 3:**
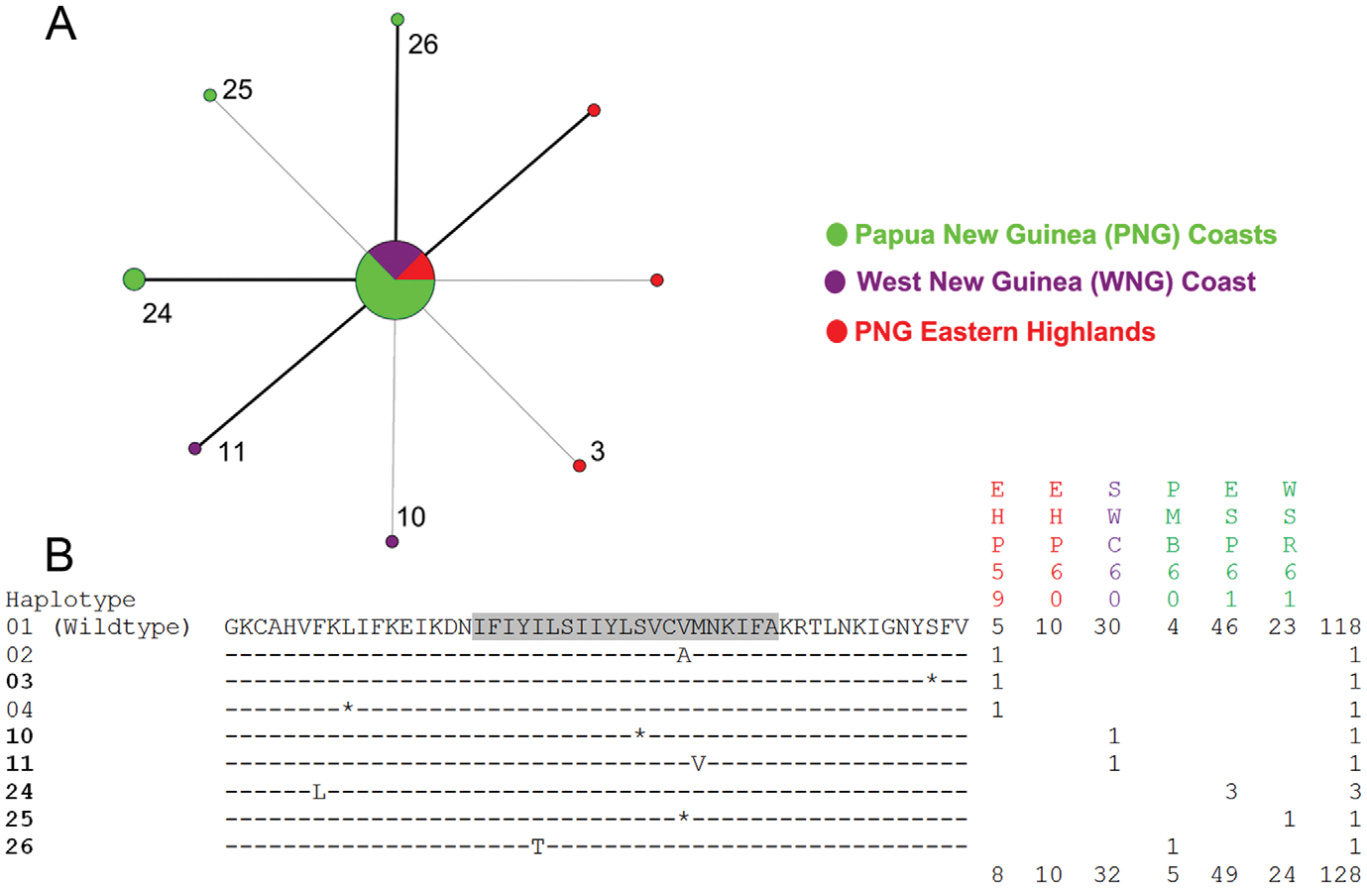
Median-joining network of *pfcrt* haplotypes (A) and alignment of translated sequences (B) from 1959–61. In the network diagram, each unique *pfcrt* haplotype is represented by a node, and the size of each node is proportional to the frequency of the haplotype. Substitutions are represented by branches, with the branch length proportional to the number of substitutions. Non-synonymous substitutions are represented by branches in bold. *Pfcrt* haplotypes that appeared in more than one time period are noted by their haplotype numbers. In the amino acid sequence alignment, the shaded region represents transmembrane domain 1. Synonymous substitutions are represented by dashes (-) and synonymous substitutions by asterisks (*). Populations are denoted by their sample codes (Table S1).

**Figure 4 pone-0030213-g004:**
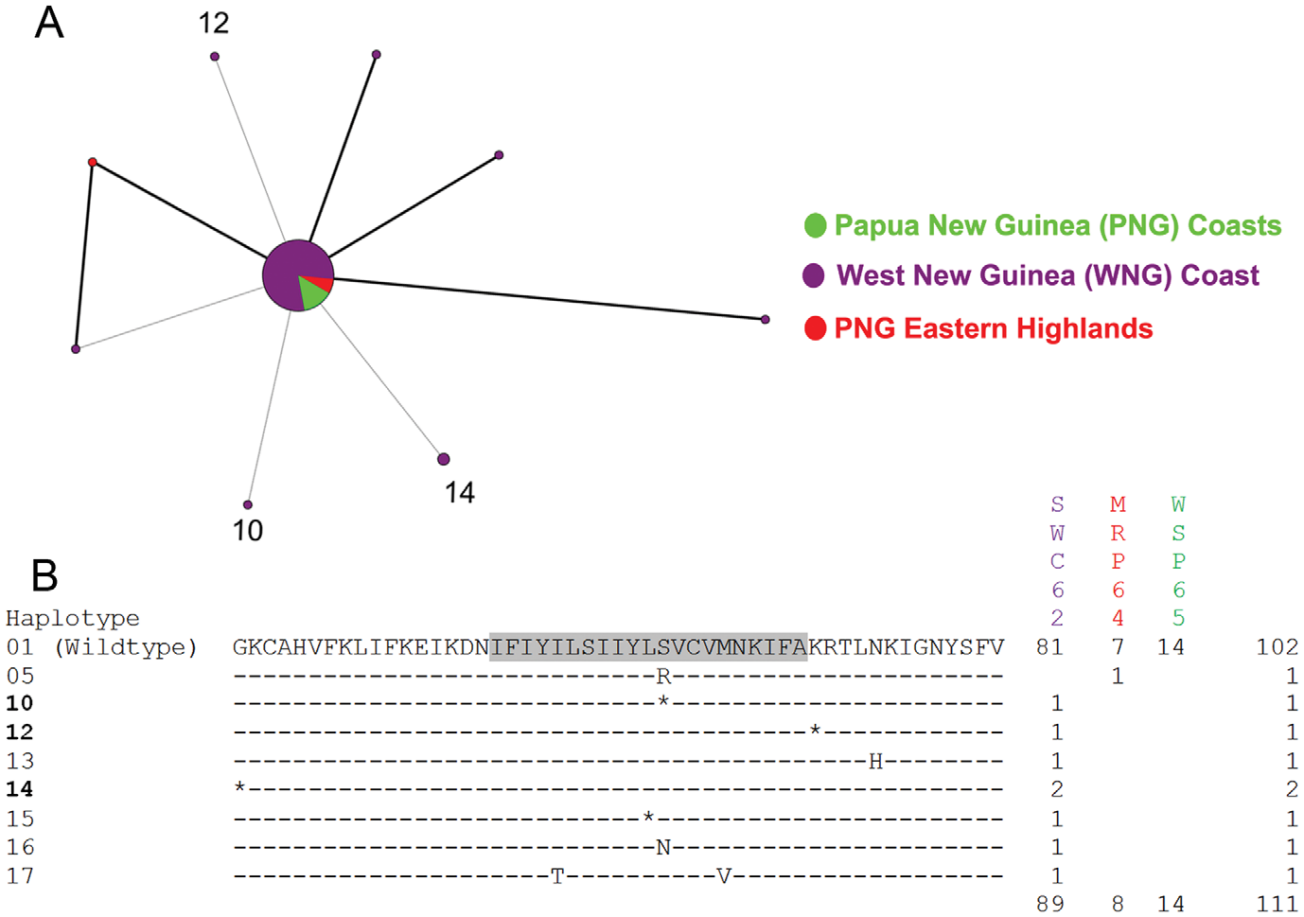
Median-joining network of *pfcrt* haplotypes (A) and alignment of translated sequences (B) from 1962–5.

**Figure 5 pone-0030213-g005:**
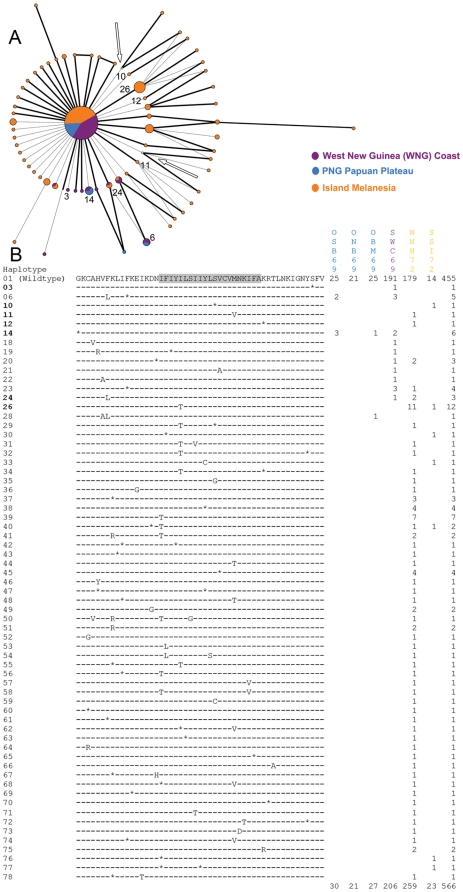
Median-joining network of *pfcrt* haplotypes (A) and alignment of translated sequences (B) from 1969–72. Hypothetical nodes are indicated by arrows.

**Figure 6:**
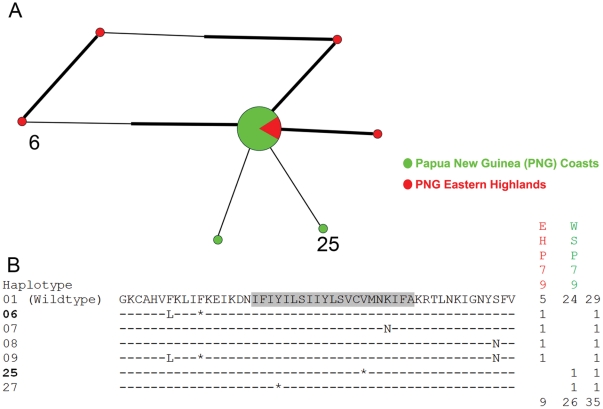
Median-joining network of *pfcrt* haplotypes (A) and alignment of translated sequences (B) from 1979.

Among the 77 mutant *pfcrt* haplotypes, non-synonymous substitutions were more abundant (34 vs. 28) and occurred more frequently (94 vs. 75) than synonymous substitutions. During the two early periods (1959–61 and 1962–65), no differences were found in the distribution of mutant *pfcrt* haplotypes among populations (Figures 3, 4). Between 1969 and 1972, significantly more (*p*<0.001) mutant *pfcrt* haplotypes were found in populations from Island Melanesia than those from the PNG Papuan Plateau and the WNG coast (Figure 5). In 1979, the PNG eastern highlands have significantly more (*p* = 0.018) mutant *pfcrt* haplotypes than the PNG coast (Figure 6). Fifty two (67.5%) mutant *pfcrt* haplotypes contained at least one non-synonymous substitution. Most mutations observed were either private or present at very low frequencies. Only five mutant haplotypes (6, 14, 24, 26, and 39) were found in five or more individuals (Figures 3–6). Three amino acid substitutions previously implicated in CQ resistance were detected in our samples. In NNH72, the N75D (codon 72–76: CVMDK) and the K76T (codon 72–76: CVMNT) substitutions were found individually in two specimens (Figure 5), while the K76N (codon 72–76: CVMNN) substitution was found in one specimen from EHP79 (Figure 6).

The relationship among *pfcrt* exon 2 haplotypes from each time period is shown by a median-joining network diagram (Figures 3–6). The wildtype *pfcrt* haplotype was shared by all populations. Nine mutant *pfcrt* haplotypes (3, 6, 10, 11, 12, 14, 24, 25, and 26) were found in samples from at least two time periods (Figures 3–6). Four of these haplotypes (6, 11, 24, and 26) harbored non-synonymous substitutions while the remaining five had only synonymous mutations (Figures 3–6). Overall, 83.3% (65/78) of haplotypes were population specific. Of the 12 mutant *pfcrt* haplotypes shared between two or more populations, 10 were shared between populations from different ecological zones.

**Figure 7:**
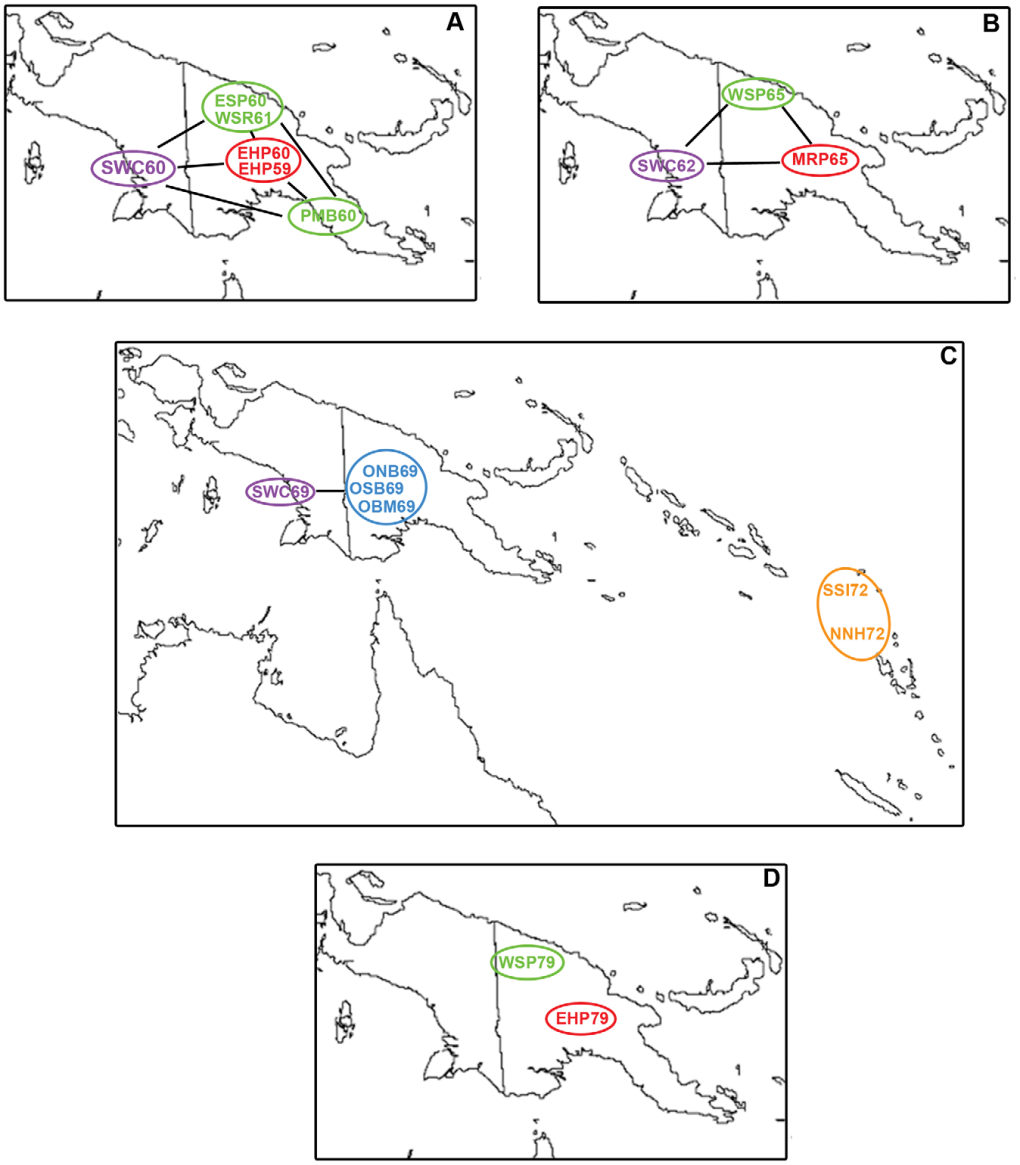
Gene flow among *P. falciparum* populations from different ecological zones. Gene flow among parasite populations from (A) 1959–61, (B) 1962–5, (C) 1969–72, and (D) 1979, is represented by solid lines. Gene flow is inferred from pairwise F_ST_ distances determined to be non-statistically significant (*p*>0.05).

We also calculated pairwise F_ST_ genetic distances to infer gene flow among parasite populations across ecological zones and persistence of parasite populations within ecological zones over time. Gene flow among populations from the same time period, as defined by non-statistically significant (*p*>0.05) pairwise F_ST_ values, is illustrated in Figure 7. During the two early periods (1959–61 and 1962–65), parasite populations from different ecological zones were not significantly differentiated from one another (Figures 7A and 7B). Between 1969 and 1972, parasite populations in Island Melanesia were significantly differentiated from those in the PNG Papuan Plateau and the WNG coast (Figure 7C). In 1979, parasites in the PNG eastern highlands were significantly differentiated from those in the PNG coast (Figure 7D). No statistically significant (*p*<0.05) F_ST_ values were found among temporally distinct samples within the same ecological region, suggesting that parasite populations within a particular region generally persisted through time. The spatial and temporal patterns of gene flow as inferred from pairwise F_ST_ distances suggested that *P. falciparum* CQ resistance in the Pacific likely arose locally and evolved in situ.

## Discussion

Malaria endemicity in the Pacific is inversely correlated with latitude and altitude [15], [16]. In PNG, malaria transmission is most intense in coastal lowlands where both temperature and rainfall remain high throughout the year, whereas in the highlands, transmission is epidemic and coincides with the conclusion of the rainy season [15]. In Vanuatu, a Y-shaped archipelago that extends along the north-south axis in the southern hemisphere, malaria transmission in the northern islands is in general more intense than in the southern islands, with the southernmost island of Aneityum being free of malaria since 1991 [16], [17]. In our archival samples, the pattern of *P. falciparum* prevalences as determined by our PCR and sequencing assays was consistent with that obtained by current malariometric surveys. Since all four commonly recognized human malaria species are present in the southwest Pacific [15], and our molecular techniques specifically targeted template DNA from *P. falciparum*, it is likely that the rates of malaria infections in these archival samples were higher than reported here, especially in the PNG highlands, where unstable malaria transmission has historically favored the predominance of *P. vivax* [18], [19].

The prevalence of *P. falciparum* infections in coastal and highland samples of New Guinea showed opposite longitudinal trends, which are consistent with changing social factors of the times. In the coastal lowlands of both PNG and WNG, the decreasing prevalence over time coincided with the initiation of malaria eradication programs. Malaria control measures began in both PNG and WNG in 1952, consisting of residual insecticide spraying, introduction of *Gambusia spp.* to swampy areas, and in some places mass drug administration of CQ and/or pyrimethamine [20]. Although complete eradication proved elusive, notable reduction in malaria prevalence was accomplished in many lowland areas despite technical and logistical difficulties [20]–[23]. In contrast to the overall decrease in *P. falciparum* prevalence in the lowlands, we observed a slight increase in infection rate in the PNG eastern highlands over time. Radford et al. [24] argued that social factors were important in changing malaria epidemiology in the highlands. Increased contact and the opening of the Highland Highways facilitated the movement of people and malaria parasites from the highly malarious coastal areas to the highlands, while many highlanders recruited as wage laborers in lowland plantations served as parasite reservoirs when they returned to the highlands. Establishment of plantations and adoption of western-style housing in the highlands also created additional mosquito breeding sites that led to increased malaria transmission [19], [24]. The increase in *P. falciparum* prevalence in our PNG eastern highland samples might also represent a gradual shift in species predominance from *P. vivax* to *P. falciparum*, as recently noted by Mueller et al. [19], although this will require molecular surveys of *P. vivax* to confirm.

In the Pacific, CQ treatment failure was first reported in WNG (Irian Jaya) in 1974 [25] and by 1980 resistance had been confirmed in all malaria endemic countries in the region [7]. Two CQ resistance-implicated amino acid substitutions, N75D and K76T were observed individually in two specimens from NNH72, resulting in the haplotypes CVMDK and CVMNT, respectively. The *pfcrt* K76T substitution has consistently been shown to correlate with CQ resistance [5], [16], [26], and the CVMNT haplotype has previously been described in CQR parasites from South America [5] and Papua [11]. The N75D substitution was associated with the K76T substitution in parasites from Cambodia [27], Thailand and Laos [28], although in the absence of the diagnostic K76T substitution, it is unclear if this CVMDK-bearing parasite had an altered response to CQ. The presence of these substitutions in parasites from northern New Hebrides in 1972 agrees with the long held suspicion that CQ resistance had been present in New Hebrides many years before it was clinically and experimentally confirmed in 1980 [29], [30].

We also detected the replacement of lysine by asparagine at *pfcrt* codon 76 in EHP79. This K76N substitution was previously experimentally induced when the CQS laboratory clone 106/1, which already had six of the seven *pfcrt* mutations found in CQR parasites from Asia and Africa, was exposed to high levels of CQ [31]. However, it is unclear whether the 76N bearing parasite we identified in this study was CQR, since it was associated with a different *pfcrt* genetic background. Whereas the mutant 106/1 parasite carried the CVIEN haplotype [31], our parasite carried the CVMNN haplotype. This rare haplotype has been reported twice in parasite isolates from Indonesia, although in both studies the in vitro response to CQ was not evaluated [28], [32].

Archival biological samples from PNG provide crucial insights into the evolution and spread of CQR parasites [13], [14], [28]. Using samples collected from 12 locations in PNG during the early 1980s, Mehlotra et al. [13] found a high frequency (97%; 33/34) of the CQR SVMNT *pfcrt* haplotype, which was consistent with a nation-wide epidemiological survey reporting widespread CQ resistance conducted during that period [33]. In contrast, the CQS CVMNK haplotype predominated (97%; 34/35) in our PNG samples collected in 1979 (EHP79 and WSP79), indicating that strong CQ selection resulted in the nearly complete replacement of CQS parasites by their resistant counterparts in as few as three years, and the latter's persistence to the present [9], [34]. It is unclear whether the CVMNN haplotype observed in our sample represented a transitional strain that ultimately gave rise to parasites carrying the more ubiquitous SVMNT haplotype seen today, or was replaced by invading parasites carrying the latter haplotype, nonetheless our data are consistent with previous interpretation that CQ resistance in the Pacific evolved *in situ* [9]. Analyses of additional samples from this critical period are currently underway and will hopefully shed light on the relationships among these haplotypes.

Of the 34 amino acid substitutions observed in our samples, 20 are considered to be non-conservative, involving a side-chain property change in either polarity and/or charge that might affect the function of the protein. Two such substitutions, I59T and I63T were observed in 14 and 19 samples respectively. In particular, mutant *pfcrt* haplotypes harboring the I63T substitution persisted through time and later dispersed into multiple regions, suggesting that these haplotypes may reflect a drug tolerant if not resistant phenotype (Figures 3–6). Transfections of CQS *P. falciparum* clones with I63T and other novel substitutions identified in this study are currently underway to evaluate their effects on CQ response.

It remains unclear how the historical use of other antimalarials prior to our earliest samples (1959) might have contributed to the selection of *pfcrt* alleles observed in this study. During WWII, malaria was a major cause of casualties for both the Japanese and the Allied troops stationed in the Southwest Pacific [35], [36]. Quinine and quinacrine, also known as mepacrine or Atabrine, were used extensively to prevent and treat malaria infections, especially among Allied soldiers [37]. The mode of action for quinine is thought to be similar to that of CQ [38], and cross resistance with CQ has been suggested [39], [40], although the association between quinine resistance in *P. falciparum* and specific substitutions in *pfcrt* has been inconclusive [41]. Quinacrine resistance was first noted in *P. falciparum* from the Wewak-Aitape region along the north coast of New Guinea in 1944 [37]. We did observe in our earliest sample from the same region (ESP61) a non-synonymous substitution (F48L) at relatively high frequency (6.1%; 3/49) (Figure 3), which might reflect previous selection by quinacrine and warrants further studies. However, it should be noted that the genetic basis for quinacrine resistance is unknown, and the use of quinacrine was largely abandoned after WWII in favor of CQ [42], such that little selective pressure was maintained during the intervening years between the end of the war and the collection of our samples.

Four factors may have contributed to the unexpectedly higher levels of *pfcrt* exon 2 genetic diversity in parasites from the PNG eastern highlands and Island Melanesia. First, in Island Melanesia the high level of parasite genetic diversity might have resulted from population structure. Samples from NNH72 and SSI72 were collected from populations on 13 and three different islands, respectively. While variable levels of travel among islands within each archipelago were noted [43], more recently extensive human contact has been essential for gene flow between parasites on different islands [44], [45]. When subdivided into individual islands, parasites on some islands were found to be genetically distinct from those on neighboring islands. For example, parasites from Gaua in northern New Hebrides were significantly distinct from those on nearby Mota Lava (F_ST_ = 0.04155; *p* = 0.03604) and Vanua Lava (F_ST_ = 0.01819; *p* = 0.03604). This implies that isolation and genetic drift on individual islands, although reducing diversity locally, have increased the genetic diversity of the archipelago as a whole.

Second, in these areas of relatively low transmission, the use of antimalarials was often reserved for treatments only [43], such that intermittent CQ pressure might have selected for a suite of different non-synonymous substitutions that only slightly altered the parasites' response to CQ. In contrast to high transmission areas where competition among different genetic strains is intense [46], even in the absence of continuous CQ selection, genetic drift may have maintained these non-synonymous substitutions in small parasite populations as suggested by low rates of infection in these low transmission areas.

Third, host immune status played an important role in the occurrence of multiple infections. In high transmission areas the host acquired immunity to malaria is constantly maintained by recurrent infections, whereas in low transmission areas malaria infections by multiple genetic strains might be more common since parasite clearance in semi-immune individuals is less effective [47]. In this study, the frequencies of multiple infections were significantly correlated with *pfcrt* haplotype diversities (R^2^ = 0.9361, *p* = 0.007), suggesting that lowered host acquired immunity in the PNG Eastern Highlands and Island Melanesia might have contributed to the high level of parasite genetic diversities in these low transmission areas.

Lastly, differences in host genetics may have affected the degree to which populations living in different ecological regions were susceptible to infection by multiple genetic strains of *P. falciparum*. Strong selection in the coastal lowland populations of PNG results in the maintenance of a number of red cell polymorphisms conferring resistance to malaria, including Southeast Asian ovalocytosis [48]–[50], alpha-thalassemia [51], beta-thalassemia [52], and Gerbich negativity [53]. It is possible that in high transmission areas, individuals with one or more of these malaria resistant alleles present a less hospitable environment to parasites, such that only those that are most adapted, i.e. either wildtype (with no *pfcrt* mutations) or fully CQR (with a complete suite of CQR *pfcrt* mutations), can survive. Conversely in low transmission areas, host selection against parasites might be less severe, thus allowing slightly less fit parasite strains, i.e. those with only one or two *pfcrt* mutations, to evolve and persist.

Genetic characterization of *P. falciparum* in the Pacific from the period of CQ resistance emergence revealed that prior to complete CQ selective sweep, *pfcrt* exon 2 was highly diverse, highlighted by a number of previously undescribed substitutions including several implicated in CQ resistance, consistent with *in situ* evolution of resistance within the Pacific. In addition to CQ selection, isolation among parasite populations and reduced strain competition in small parasite populations, combined with lowered acquired immunity and genetic resistance to malaria among hosts in low transmission areas, might have been crucial to the evolution and initial establishment of CQR *P. falciparum* in the Pacific. These data suggest that populations at the peripheries of malaria endemic areas may be the most crucial to monitor for parasites evolving tolerance and resistance to currently used antimalarials.

## Materials and Methods

### Ethics Statement

This study was submitted to the Human Subjects Research Review Committee of Binghamton University, where it was reviewed and determined exempt because no human DNA was examined (Protocol # 787-08).

### Archival Human Serum Collections

Samples used in this study were provided by the Binghamton University Serum Archive Facility at Binghamton, NY, USA, and their use in this study was approved by the institutional review board of Binghamton University. Samples were collected under the ethical and legal guidelines of the United States National Institutes of Health and all existing international standards of the time, and their continued use for biomedical research has recently been reaffirmed by the Papua New Guinea Medical Research Advisory Committee (documentation available upon request). A total of 4598 specimens from 19 Pacific populations sampled between 1959 and 1979 was included in this study. The locations of sample collections and brief descriptions are given in figure 1 and table S1, respectively. Samples collected from the same locations in different years did not represent repeated samplings of the same populations.

### Nucleic Acid Extraction

DNA was extracted from 200 µl of human serum using either the QIAamp Blood Mini Kit (QIAgen Biosciences, Germantown, MD) or the automated Maxwell-16 system (Promega, Madison, WI) according to the respective manufacturers' instructions. Eluted DNA was frozen at −20°C until use.

### Molecular Analysis

A segment of the *pfcrt* exon 2 was PCR-amplified in a 12.5 µl reaction containing 4 µl of DNA, 0.2 mM of dNTP, 2 mM of MgCl_2_, 0.5 µM of each primer (CRT76-sense 5′-GGTGGAGGTTCTTGTCTTGG-3′ and CRT76-antisense 5′-ATAAAGTTGTGAGTTTCGGATG-3′) [54], and 0.25 unit of HotStar *Taq* DNA polymerase (QIAgen Biosciences, Germantown, MD). The cycling conditions consisted of 15 minutes at 95°C, 90 seconds at 61°C, 60 seconds at 72°C for 1 cycle; 10 seconds at 95°C, 90 seconds at 61°C, and 2 minutes at 72°C for 50 cycles, and a final extension for 10 minutes at 72°C. PCR amplicons were purified using the Millipore Manu03050 Filter Plate (Millipore, Billerica, MA), and were sequenced in both directions using internal primers, CRTD1 5′-TGTGCTCATGTGTTTAAACTT-3′ and CRTD2 5′-CAAAACTATAGTTACCAATTTTG-3′ [55], with the BigDye Terminator Kit v.3.1 on an ABI 3730xl DNA Analyzer (Applied Biosystems, Foster City, CA).

Electropherograms were visually checked using Sequencing Analysis 2.1 (Applied Biosystems, Foster City, CA). Infection by genetically distinct *P. falciparum* strains was inferred when heterologous peaks were present in a single specimen. In cases where heterologous peaks were observed at more than one nucleotide position in a single specimen, *pfcrt* haplotypes were constructed based on the relative peak heights at each nucleotide position in question. Assuming that multiple substitutions in a lineage arose in a stepwise manner, we examined the position of the constructed halpotypes in the network diagrams (Figures 3–6) [56]. Only two branches directly stemming from the wildtype *pfcrt* haplotype contained hypothetical internal nodes (Figure 5), indicating that most of these constructed haplotypes were derived from simpler haplotypes that were also present in our data set.

### Statistical Analyses

Prevalence of *P. falciparum* infection was determined at two hierarchical levels. Since malaria endemicity in the Pacific varies by altitude and latitude [15], [16], in addition to comparisons among all samples, individual samples were assigned to one of five ecological zones: (1) PNG eastern highlands, (2) WNG coast, (3) PNG coasts, (4) PNG Papuan Plateau, and (5) Island Melanesia, consisting of New Hebrides (now Vanuatu) and Solomon Islands (Figure 1). Samples used in this study were collected over a span of 20 years (1959–79), and were therefore divided into the following periods, (A) 1959–61, (B) 1962–5, (C) 1969–72, and (D) 1979, to account for temporal variation and assess the persistence of genotypes over time.

*Pfcrt* haplotype diversity was calculated for each population and each ecological zone using the equation: H = n(1−∑X_i_^2^)/(n−1), where n is the number of *pfcrt* sequences, and X_i_ is the frequency of the i-th haplotype [57]. Mean *pfcrt* haplotype diversities among ecological zones were compared using one-way analysis of variance (ANOVA). For each time period, *G*-test for homogeneity was used to compare the distribution of mutant *pfcrt* haplotypes among populations. Median-joining network diagrams were constructed using the software Network 4.600 to illustrate the phylogenetic relationships among all *pfcrt* sequences for each period [56], [58].

To infer gene flow among populations, two sets of pairwise F_ST_ genetic distances were calculated using Arlequin 3.1 [59]. The first set consisted of comparisons of samples from the same time period to examine gene flow across ecological zones, while the second consisted of samples from the same ecological zone but were collected in different periods to infer replacement (or persistence) over time. The statistical significance (*p*<0.05) of the observed values was evaluated by randomly permuting sequences among populations approximately 10,000 times to generate a null distribution against which the observed values were compared.

## Supporting Information

Table S1 The approximate geographic locations of these samples are shown in Figure 1. Samples collected from the same locations in different years did not represent repeated samplings of the same population. Ages of donors for the sample EHP79 were not available.
(DOC)

## References

[1] World Health Organization (2009). World malaria report 2009.

[2] Trape JF (2001). The public health impact of chloroquine resistance in Africa.. Am J Trop Med Hyg.

[3] Wongsrichanalai C, Pickard AL, Wernsdorfer WH, Meshnick SR (2002). Epidemiology of drug-resistant malaria.. Lancet Infect Dis.

[4] Noedl H, Se Y, Schaecher K, Smith BL, Socheat D (2008). Evidence of artemisinin-resistant malaria in western Cambodia.. N Engl J Med.

[5] Fidock DA, Nomura T, Talley AK, Cooper RA, Dzekunov SM (2000). Mutations in the *P. falciparum* digestive vacuole transmembrane protein PfCRT and evidence for their role in chloroquine resistance.. Mol Cell.

[6] Wootton JC, Feng X, Ferdig MT, Cooper RA, Mu J (2002). Genetic diversity and chloroquine selective sweeps in *Plasmodium falciparum*.. Nature.

[7] Mita T, Kaneko A, Hombhanje F, Hwaihwanje I, Takahashi N (2006). Role of *pfmdr1* mutations on chloroquine resistance in *Plasmodium falciparum* isolates with *pfcrt* K76T from Papua New Guinea.. Acta Trop.

[8] Yung AP, Bennett NM (1976). Chloroquine-resistant *falciparum* malaria acquired in Papua New Guinea.. Med J Aust.

[9] Mehlotra RK, Fujioka H, Roepe PD, Janneh O, Ursos LM (2001). Evolution of a unique *Plasmodium falciparum* chloroquine-resistance phenotype in association with *pfcrt* polymorphism in Papua New Guinea and South America.. Proc Natl Acad Sci USA.

[10] Anderson TJ, Haubold B, Williams JT, Estrada-Franco JG, Richardson L (2000). Microsatellite markers reveal a spectrum of population structures in the malaria parasite *Plasmodium falciparum*.. Mol Biol Evol.

[11] Nagesha HS, Casey GJ, Rieckmann KH, Fryauff DJ, Laksana BS (2003). New haplotypes of the *Plasmodium falciparum* chloroquine resistance transporter (*pfcrt*) gene among chloroquine-resistant parasite isolates.. Am J Trop Med Hyg.

[12] Hastings IM, Watkins WM (2005). Intensity of malaria transmission and the evolution of drug resistance.. Acta Trop.

[13] Mehlotra RK, Mattera G, Bhatia K, Reeder JC, Stoneking M (2005). Insight into the early spread of chloroquine-resistant *Plasmodium falciparum* infections in Papua New Guinea.. J Infect Dis.

[14] Chan CW, Lynch D, Spathis R, Hombhanje FW, Kaneko A (2006). Flashback to the 1960s: utility of archived sera to explore the origin and evolution of *Plasmodium falciparum* chloroquine resistance in the Pacific.. Acta Trop.

[15] Müller I, Bockarie M, Alpers M, Smith T (2003). The epidemiology of malaria in Papua New Guinea.. Trends Parasitol.

[16] Kaneko A, Taleo G, Kalkoa M, Yaviong J, Reeve PA (1998). Malaria epidemiology, glucose 6-phosphate dehydrogenase deficiency and human settlement in the Vanuatu Archipelago.. Acta Trop.

[17] Kaneko A, Taleo G, Kalkoa M, Yamar S, Kobayakawa T (2000). Malaria eradication on islands.. Lancet.

[18] Mueller I, Bjorge S, Poigeno G, Kundi J, Tandrapah T (2003). The epidemiology of malaria in the Papua New Guinea highlands: 2. Eastern Highlands Province.. P N G Med J.

[19] Mueller I, Namuigi P, Kundi J, Ivivi R, Tandrapah T (2005). Epidemic malaria in the highlands of Papua New Guinea.. Am J Trop Med Hyg.

[20] Spencer M (1992). The history of malaria control in the southwest Pacific region, with particular reference to Papua New Guinea and the Solomon Islands.. P N G Med J.

[21] Metselaar D (1961). Seven years' malaria research and residual house spraying in Netherlands New Guinea.. Am J Trop Med Hyg.

[22] Peters W (1962). A critical survey of the results of malaria-eradication and control programmes in the Southwest Pacific.. Ann Trop Med Parasitol.

[23] Smithurst BA (1970). A malaria survey on Bougainville Island (Kieta Subdistrict) January–February 1967.. Southeast Asian J Trop Med Public Health.

[24] Radford AJ, Van Leeuwen H, Christian SH (1976). Social aspects in the changing epidemiology of malaria in the highlands of New Guinea.. Ann Trop Med Parasitol.

[25] Clyde DF, McCarthy VC, Miller RM, Hornick RB (1976). Chloroquine-resistant *falciparum* malaria from Irian Jaya (Indonesian New Guinea).. J Trop Med Hyg.

[26] Durand R, Jafari S, Vauzelle J, Delabre JF, Jesic Z (2001). Analysis of *pfcrt* point mutations and chloroquine susceptibility in isolates of *Plasmodium falciparum*.. Mol Biochem Parasitol.

[27] Lim P, Chy S, Ariey F, Incardona S, Chim P (2003). *pfcrt* polymorphism and chloroquine resistance in *Plasmodium falciparum* strains isolated in Cambodia.. Antimicrob Agents Chemother.

[28] Saito-Nakano Y, Tanabe K, Kamei K, Iwagami M, Komaki-Yasuda K (2008). Genetic evidence for *Plasmodium falciparum* resistance to chloroquine and pyrimethamine in Indochina and the Western Pacific between 1984 and 1998.. Am J Trop Med Hyg.

[29] Bowden DK, Bastien P, Douglas FP, Muir JW, Tambisari E (1982). Chloroquine-reistant *Plasmodium falciparum* malaria in Vanuatu.. Med J Aust.

[30] Parkinson AD, Bowden DK (1980). Chloroquine resistance in the south-west Pacific.. Med J Aust.

[31] Cooper RA, Ferdig MT, Su XZ, Ursos LM, Mu J (2002). Alternative mutations at position 76 of the vacuolar transmembrane protein PfCRT are associated with chloroquine resistance and unique stereospecific quinine and quinidine responses in *Plasmodium falciparum*.. Mol Pharmacol.

[32] Huaman MC, Yoshinaga K, Suryanatha A, Suarsana N, Kanbara H (2004). Short report: polymorphisms in the chloroquine resistance transporter gene in *Plasmodium falcipaum* isolates from Lombok, Indonesia.. Am J Trop Med Hyg.

[33] Dulay IS, Gibson FD, Eyeson-Annan MB, Narara A (1987). Chloroquine resistance in *Plasmodium falciparum* and its geographical distribution in Papua New Guinea.. P N G Med J.

[34] Mita T, Kaneko A, Hombhanje F, Hwaihwanje I, Takahashi N (2006). Role of *pfmdr1* mutations on chloroquine resistance in *Plasmodium falciparum* isolates with *pfcrt* K76T from Papua New Guinea.. Acta Trop.

[35] Sweeney AW (2000). Wartime research on malaria chemotherapy.. Parassitologia.

[36] Joy RJ (1999). Malaria in American troops in the South and Southwest Pacific in World War II.. Med Hist.

[37] Sweeney AW (1996). The possibiligy of an “X” factor. The first documented drug resistance of human malaria.. Int J Parasitol.

[38] Warhurst DC (1981). The quinine-haemin interaction and its relationship to antimalarial activity.. Biochem Pharmacol.

[39] Simon F, Le Bras J, Charmot G, Girard PM, Faucher C (1986). Severe chloroquine-resistant falciparum malaria in Gabon with decreased sensitivity to quinine.. Trans R Soc Trop Med Hyg.

[40] Warsame M, Wernsdorfer WH, Payne D, Björkman A (1991). Susceptibility of *Plasmodium falciparum* in vitro to chloroquine, mefloquine, quinine and sulfadoxine/pyrimethamine in Somalia: relationships between the responses to the different drugs.. Trans R Soc Tom Med Hyg.

[41] Okombo J, Ohuma E, Picot S, Nzila A (2011). Update on genetic markers of quinine resistance in *Plasmodium falciparum*.. Mol Biochem Parasitol.

[42] Peters W, Peters W, Richards WHG (1984). History and current status of drug resistance.. Antimalarial Durgs: Biological Background, Experimental Methods, and Drug Resistance.

[43] Brown P, Collins WE, Gajdusek DC, Miller LH (1976). An evaluation of malaria fluorescent antibody patterns in several remote island populations of the New Hebrides, Solomons, Western Carolines, and New Guinea.. Am J Trop Med Hyg.

[44] Lum JK, Kaneko A, Tanabe K, Takahashi N, Björkman A (2004). Malaria dispersal among islands: human mediated *Plasmodium falciparum* gene flow in Vanuatu, Melanesia.. Acta Trop.

[45] Lum JK, Kaneko A, Taleo G, Amos M, Reiff DM (2007). Genetic diversity and gene flow of humans, *Plasmodium falciparum*, and *Anopheles farauti s.s.* of Vanuatu; inferred malaria dispersal and implications for malaria control.. Acta Trop.

[46] Mita T, Kaneko A, Lum JK, Zungu IL, Tsukahara T (2004). Expansion of wild type allele rather than back mutation in *pfcrt* explains the recent recovery of chloroquine sensitivity of *Plasmodium falciparum* in Malawi.. Mol Biochem Parasitol.

[47] Langhorne J, Ndungu FM, Sponaas AM, Marsh K (2008). Immunity to malaria: more questions than answers.. Nat Immunol.

[48] Allen SJ, O'Donnell A, Alexander ND, Mgone CS, Peto TE (1999). Prevention of cerebral malaria in children in Papua New Guinea by Southeast Asian ovalocytosis band 3.. Am J Trop Med Hyg.

[49] Genton B, al-Yaman F, Mgone CS, Alexander N, Paniu MM (1995). Ovalocytosis and cerebral malaria.. Nature.

[50] Mgone CS, Koki G, Paniu MM, Kono J, Bhatia KK (1996). Occurrence of the erythrocyte band 3 (AE1) gene deletion in relation to malaria endemicity in Papua New Guinea.. Trans R Soc Trop Med Hyg.

[51] Flint J, Hill AV, Bowden DK, Oppenheimer SJ, Sill PR (1986). High frequencies of alpha-thalassaemia are the result of natural selection by malaria.. Nature.

[52] Hill AVS, Bowden DK, O'Shaughnessy DF, Weatherall DJ, Clegg JB (1988). Beta thalassemia in Melanesia: association with malaria and characterization of a common variant (IVS-1 nt 5 G—C).. Blood.

[53] Serjeantson SW (1989). A selective advantage for the Gerbich-negative phenotype in malarious areas of Papua New Guinea.. PNG Med J.

[54] Ariey F, Fandeur T, Durand R, Randrianarivelojosia M, Jambou R (2006). Invasion of Africa by a single *pfcrt* allele of South East Asian type.. Malar J.

[55] Djimdé A, Doumbo OK, Cortese JF, Kayentao K, Doumbo S (2001). A molecular marker for chloroquine-resistant *falciparum* malaria.. N Engl J Med.

[56] Network 4.516.. http://www.fluxus-engineering.com.

[57] Nei M (1987). Molecular Evolutionary Genetics.

[58] Bandelt HJ, Forster P, Röhl A (1999). Median-joining networks for inferring intraspecific phylogenies.. Mol Biol Evol.

[59] Excoffier L, Laval G, Schneider S (2005). Arlequin (version 3.0): An integrated software package for population genetics data analysis.. Evol Bioinform Online.

