# Supplementary material for: Diversity of Plasmodium falciparum Chloroquine Resistance Transporter (pfcrt) Exon 2 Haplotypes in the Pacific from 1959 to 1979
Source: PLoS One. 2012 Jan 17;7(1):e30213. doi: 10.1371/journal.pone.0030213 (PMC3260253; doi:10.1371/journal.pone.0030213)
Supplement: Table S1 — The approximate geographic locations of these samples are shown in Figure 1. Samples collected from the same locations in different years did not represent repeated samplings of the same population. Ages of donors for the sample EHP79 were not available. (DOC) [file pone.0030213.s001.doc]

Table S1: Prevalence of *P. falciparum* infections and *pfcrt* exon 2 haplotype diversity for sampled populations and ecological zones.

| **Ecological Zone** | **Sample Code** | **Location** | **Altitude (m)** | **Year** | **Number of Specimens** | **Average Age** | **Number Infected (%)** | **Multiple Infection (%)** | **Number of *pfcrt* Sequences** | **Haplotype Diversity (+/- SD)** |
| --- | --- | --- | --- | --- | --- | --- | --- | --- | --- | --- |
| Papua New Guinea (PNG) Eastern Highlands | EHP59 | Eastern Highland Province | 900-1500 | 1959 | 115 | 21.2 | 6 (5.22) | 2 (33.3) | 8 | 0.643 (0.184) |
|  | EHP60 | Eastern Highland Province | 900-1500 | 1960 | 604 | 21.8 | 10 (1.66) | 0 (0.00) | 10 | 0.000 (0.000) |
|  | BAR62 | Baira | 1700 | 1962 | 30 | 29.2 | 0 (0.00) | 0 (0.00) | 0 | N/D |
|  | MRP64 | Morobe Province | 1400 | 1964 | 121 | 20.5 | 7 (5.79) | 1 (14.3) | 8 | 0.250 (0.180) |
|  | EHP79 | Eastern Highland Province | 2100 | 1979 | 86 | N/A | 7 (8.14) | 2 (28.6) | 9 | 0.722 (0.159) |
|  |  |  |  | Subtotal | 956 |  | 30 (3.14) | 5 (16.7) | 35 | 0.410 (0.106) |
| West New Guinea (WNG) Coast | SWC60 | Southwest Coast | 0-5 | 1960 | 56 | 10.6 | 31 (55.4) | 1 (3.23) | 32 | 0.123 (0.078) |
|  | MRK61 | Merauke | 5-10 | 1961 | 39 | 15.4 | 0 (0.00) | 0 (0.00) | 0 | N/D |
|  | SWC62 | Southwest Coast | 0-5 | 1962 | 437 | 18.8 | 87 (19.9) | 2 (2.30) | 89 | 0.172 (0.054) |
|  | SWC69 | Southwest Coast | 0-5 | 1969 | 644 | 20.5 | 198 (30.7) | 8 (4.04) | 206 | 0.140 (0.033) |
|  |  |  |  | Subtotal | 1176 |  | 316 (26.9) | 11 (3.48) | 327 | 0.147 (0.027) |
| PNG Coasts | PMB60 | Port Moresby | 10-50 | 1960 | 35 | 6.8 | 5 (14.3) | 0 (0.00) | 5 | 0.400 (0.237) |
|  | ESP61 | East Sepik Province | 0-5 | 1961 | 146 | 28.3 | 46 (31.5) | 3 (6.52) | 49 | 0.117 (0.060) |
|  | WSR61 | Wosera | 5-10 | 1961 | 66 | 15.8 | 24 (36.4) | 0 (0.00) | 24 | 0.083 (0.075) |
|  | WSP65 | West Sepik Province | 100-130 | 1965 | 59 | 20.3 | 14 (23.7) | 0 (0.00) | 14 | 0.000 (0.000) |
|  | WSP79 | West Sepik Province | 100-160 | 1979 | 255 | 25.7 | 25 (9.80) | 1 (0.04) | 26 | 0.151 (0.093) |
|  |  |  |  | Subtotal | 561 |  | 114 (20.3) | 4 (3.51) | 118 | 0.115 (0.040) |
| PNG Papuan Plateau | OSB69 | Olsobip | 450-550 | 1969 | 101 | 16.5 | 28 (27.7) | 2 (7.14) | 30 | 0.301 (0.102) |
|  | ONB69 | Onabasulu | 1000-1200 | 1969 | 231 | 23.0 | 21 (9.09) | 0 (0.00) | 21 | 0.000 (0.000) |
|  | OBM69 | Obeimi | 1500 | 1969 | 218 | 23.3 | 26 (11.9) | 1 (3.85) | 27 | 0.145 (0.090) |
|  |  |  |  | Subtotal | 550 |  | 75 (13.6) | 3 (4.00) | 78 | 0.170 (0.057) |
| Island Melanesia | NNH72 | Northern New Hebrides | 0-50 | 1972 | 1158 | 16.0 | 216 (18.7) | 43 (19.9) | 259 | 0.520 (0.039) |
|  | SSI72 | Southern Solomon Islands | 0-50 | 1972 | 197 | 16.6 | 20 (10.2) | 3 (15.0) | 23 | 0.585 (0.122) |
|  |  |  |  | Subtotal | 1355 |  | 236 (17.4) | 46 (19.5) | 282 | 0.525 (0.037) |
|  |  |  |  | Total | 4598 |  | 771 (16.8) | 69 (8.95) | 840 | 0.295 (0.021) |
